# Cultural and religious determinants of HIV transmission: A qualitative study with people living with HIV in Belu and Yogyakarta, Indonesia

**DOI:** 10.1371/journal.pone.0257906

**Published:** 2021-11-15

**Authors:** Nelsensius Klau Fauk, Paul Russell Ward, Karen Hawke, Lillian Mwanri

**Affiliations:** 1 College of Medicine and Public Health, Flinders University, Adelaide, South Australia, Australia; 2 Institute of Resource Governance and Social Change, Kupang, Nusa Tenggara Timur, Indonesia; 3 Infectious Disease—Aboriginal Health, South Australian Health and Medical Research Institute, Adelaide, Australia; Georgia Southern University, UNITED STATES

## Abstract

As a part of a larger qualitative study to understand HIV-risk factors and impacts on people living with HIV (PLHIV) (52 women and 40 men) in Belu and Yogyakarta, Indonesia, this paper reports the influences of cultural practices and religious beliefs on sexual relationships and behaviours of participants as contributors for HIV transmission. This study was conducted from June to December 2020. Data collection was conducted using one-one-one in-depth interviews. Participants were recruited using the snowball sampling technique. Data analysis was guided by a qualitative data analysis framework. The findings showed that cultural practices in Belu related to the use of bride wealth, managing spousal disputes, marriage, and condom use, influenced spousal relationships and sexual behaviours or practices which contributed to HIV transmission. Javanese cultural practices and expectation of an ‘ideal wife’, Islamic religious beliefs about expected husband-wife relationships, forbidden premarital sex, and the participants’ individual interpretation of their religious beliefs about condom use spousal sexual relations, also influenced spousal sexual relations and behaviours, which supported HIV transmission among the participants. The findings indicate the need for HIV education programs that address cultural practices and religious beliefs for community members and population groups to enhance their understanding about HIV, condom use, and how cultural practices and religious beliefs play a role in HIV transmission. The findings also indicate the need for involvement of religious leaders in HIV education programs to bring insights to people and help them interpret their religious beliefs in health promoting ways. Future studies that explore different aspects of culture and religion which may contribute to HIV transmission are recommended.

## Introduction

The 2020 UNAIDS report [1] showed a global estimation of 38 million people living with HIV (PLHIV) and 1.7 million newly diagnoses in 2019. The same report showed a reduction in new HIV infections of 12% and 29% less AIDS-related deaths during the period from 2010 to 2019, both of which are associated with the coverage expansion and effectiveness of antiretroviral therapy [1]. However, the UNAIDS report also showed that HIV infection in the context of Indonesia increased by 25.5% during the period from 2010 to 2018, with an estimated 510,000 cases in 2010 to 640,000 in 2018 [2]. Behavioural factors such as unprotected sexual intercourse and injecting drug use (IDU) have been reported as the main routes of HIV transmission in the country [3].

Globally, behavioural factors such as frequent engagement in unprotected sex or sex without condoms with multiple partners [4–8] and IDU involving needle sharing [9, 10] have been well-documented as the main contributors for HIV transmission. Low level of education, lack of knowledge of the means of HIV transmission and prevention, unavailability of condoms in the moment of need [11–14] and lack of clean needles or syringes [9] are reported as the supporting factors for people’s engagement in such high-risk behaviours. Poor economic or financial conditions is another contributor for HIV transmission as it is reported to often lead to people’s involvement in transactional sex or sex with multiple partners in exchange for money and the experience of sexual coercion which often occurs without protection or condom use due to refusal by sex clients or partners [15–17]. Social environmental factors such as availability and ease of accessibility of brothels and peer social influence on casual sex practices and poor condom use have also been as attributable to HIV transmission within population groups or communities [13, 18–22]. A study in Cambodia also reported an increase in HIV transmission within couples due to cultural practices where a woman or wife has a duty to sexually serve her husband and is expected to accept her husband’s promiscuity [23]. This is tied in with cultural practices where men or husbands are perceived to be entitled to sex and accepted to have multiple sex partners, while women are expected to remain faithful, fulfil their duty and submit to their husbands [24–26].

Although many studies globally have explored HIV-risk factors among different population groups, the majority have focused on individual, structural and socioeconomic-related factors [27–30]. Similarly, studies on HIV-risk factors in the context of Indonesia have mainly covered individual level factors (e.g., knowledge of HIV, sexual behaviours, condom use, IDU, environmental and socioeconomic factors (e.g., peer influence on risky behaviours, surrounding environment and poor economic condition that facilitate people’s engagement risky behaviours) [8, 13, 31–33]. Evidence of cultural and religious factors that contribute to HIV transmission is still limited, despite 87.18% and 9.87% of the Indonesian population following Islam and Christianity respectively [34]. This paper fills this gap and enriches the existing knowledge of cultural and religious-related determinants of HIV transmission by presenting an analysis of the influence of cultural practices and religious beliefs on sexual relations, condom use practices and sexual behaviours of women and men living with HIV in Belu and Yogyakarta, Indonesia. Indonesia is a country where most people actively follow religion, and hold firmly beliefs and values that are influenced by religious and cultural norms. These have a significant influence on daily life, attitudes, behaviours and relationships [35, 36], so understanding religious and cultural factors is critical for the development of HIV programs and interventions. This understanding can improve knowledge, health promoting attitudes and behaviours of PLHIV and general community members in the study settings and other settings in Indonesia and globally.

### Theoretical framework

The logical model for behavioural and environmental factors diagnosis was used to conceptualise and discuss the findings of the current study [37]. This model suggests that socioenvironmental factors, including social influences, cultural norms, values and practice, determine or contribute to health problem among people within population groups and communities [37, 38]. These aspects determine health problem through their influence on people’s behaviours, such as sexual behaviours and condom use practices. Therefore, health behaviour interpretations should also be based on people’s perceptions on behaviours and the influence of socioenvironmental factors that nurture these behaviours [37]. This model also suggests that personal factors can also influence people’s sexual behaviours and condom use practices, which are supporting HIV transmission among them [39].

## Methods

The report of the methods section was guided by consolidated criteria for reporting qualitative studies (COREQ) checklist (S1 Checklist) (40). The checklist contains 32 items that need to be covered to support the explicit and comprehensive reporting of qualitative studies [40].

### Study setting

This study was conducted in Belu and Yogyakarta from June to December 2019. Belu is a part of East Nusa Tenggara (Nusa Tenggara Timor) province, located in the Eastern part of Indonesia, and shares a border with East Timor. Belu district consists of 12 sub-districts and has a total population of 204,541 people including 100,922 male and 103,619 female [41]. The majority of people in Belu are from several tribes, including Tetun, Marae, Kemak, Atoni dan Rote. There are several local languages, in addition to Bahasa (the Indonesian national language), spoken by people in Belu, such as Tetun, Bunaq, Kemak dan Dawan [41]. In terms of healthcare facilities, Belu has a public hospital, and two private hospitals, 17 community health centres, 21 sub-community health centres, 48 village maternity posts, 23 village health posts and 5 private clinics [41]. It only has one HIV clinic providing HIV treatment or antiretroviral therapy (ART) for HIV positive patients. Yogyakarta city is part of the Special Region of Yogyakarta province. This city has a total population of 636,660 people and consists of 14 sub-districts with the coverage area of 32.50 km^2^ [42]. The populations in Yogyakarta are from different ethnic groups, such as Java (the majority), Sunda, Melayu, Thionghoa, Batak, Mingkabau, Bali, Madura, Banjar, Bugis, Betawi and Banten [42]. Javanese, in addition to Bahasa, is the language widely spoken in Yogyakarta [43]. In terms of healthcare facilities, it has two government hospitals, 18 private hospitals, 18 public health centres and nine sub-public health centres [44]. Of these healthcare facilities, four hospitals and 10 community health centres provide HIV care services for HIV positive patients [3, 45].

There is no significant differences in the number of HIV cases in the two settings, with Belu reporting 1,200 HIV cases and Yogyakarta reporting 1,353 cases [46, 47]. Previous studies have reported several factors that have contributed to HIV transmission across both settings [8, 13, 14, 18, 48], but aspects of culture and religion have not been explored before. Belu and Yogyakarta are different in regards to religion perspectives with Belu being a traditionally Christian area where the majority of people hold Timorese culture, while Yogyakarta municipality is a traditionally Muslim area with the majority of people holding Javanese culture. The differences in religion or religious beliefs and cultural practices about sex, condom use, spousal (sexual) relationships, the position of a wife in a marriage, etc, were part of the reasons for the choice for these two study settings. The choice to include these two settings in this study provided opportunities for the researchers to explore and understand whether or not and how these aspects played a role in the transmission of HIV among the participants. To best of our knowledge, evidence on cultural and religious factors that contribute to HIV transmission is still limited in the context of Indonesia and due to feasibility, familiarity and the potential of undertaking the current study successfully, Yogyakarta and Belu were selected as the study settings.

### Study design and recruitment of the participants

Data reported in this paper are part of a large-scale study that aimed to understand HIV risk factors and impacts on PLHIV and their families in Belu and Yogyakarta, Indonesia. This study used a qualitative methodology exploring perceptions, understandings and interpretations about factors that may have played a role in the transmission of HIV among participants, their experiences of the challenges of living with HIV, and their access to HIV healthcare services [49].

The recruitment of the participants employed snowball sampling technique. Initially, the researcher solicited the help from the receptionists at the HIV clinics in both study settings to distribute the study information sheets containing the researchers’ contact details to patients with HIV who used their services. Patients with HIV who called and stated their intention to be involved in the interview were recruited and asked to recommend a preferred time and place for the interview. After interviews each participant was also asked to distribute the information sheets to their eligible friends and colleagues who might be willing to participate in the study. This process was recursive and took a duration of six months. Only two potential participants withdrew their participations due to personal reasons and finally 92 PLHIV comprising 46 from each setting (52 women and 40 men) aged 18 years old or above participated in the study. Recruitment of participants in Yogyakarta ceased after interviewing 46 participants because of data saturation and we then wanted to interview the same number in Belu for consistency, but data saturation was also met in Belu by the 46^th^ participant.

### Data collection

Data collection employed in-depth interviews which were conducted in a private room at the HIV clinic in Belu and in a rented house close to the HIV clinic in Yogyakarta, with only the researcher and each participant in the room. The interviews were conducted by the first author (male PhD student), and it should be acknowledged that there were no restrictions on inter-sex interactions among Muslims in Yogyakarta. Interviews were carried out in Bahasa, the national language of Indonesia, which is spoken in both Belu and Yogyakarta and the primary language of the researcher, who also speaks fluent English. The researcher has attended formal training in qualitative methods and regularly conducted research on public health issues, including HIV/AIDS. Interviews were digitally audio recorded and fieldnotes were also undertaken by the researcher once felt necessary. The duration of the interviews varied ranging from 35 to 87 minutes, with the total of 4,827 minutes for 92 in-depth interviews. None of the participants were known to the researcher prior to the data collection. An interview guide was prepared and used during the interviews (S1 File). The topics on cultural and religious aspects explored in this study focused on several main areas, including practices and beliefs in the participants’ culture and religion about husband-and-wife relationships in marriage, extramarital and premarital sex, and how these influenced their sexual relations. Similarly, we explored the participants’ cultural practices and religious beliefs about the purpose of marriage, sex and condom use and how these influenced their sexual relations and condom use behaviours. We also explored participants’ views and experiences of cultural and religious-related factors that shaped or influenced their sexual relations and practices. Data collection from each group of female and male participants in each setting stopped when the researchers felt that data had been rich enough to address the study objective and data saturation had been reached. Data saturation in each group was indicated in the information or responses provided by the last few participants, which were similar to those of previous participants. Interviews started with the female and male groups in Yogyakarta and continued with the other groups in Belu. We did not offer an opportunity for participants to read and correct the information provided after the transcription due to the sensitivity of the topic and to prevent the possibility of the transcripts being received and read by their family members, which might divulge the participants’ HIV status, in case they had not disclosed it to family members. No repeated interview was conducted.

### Data analysis

The first author (NKF) transcribed the recorded interviews manually in Bahasa and then the transcripts were imported to NVivo 12 for analysis. Coding and analysis were performed in Bahasa to retain the cultural, religious and social meanings of the data or information from the participants [50]. For the purpose of publication, quotes were translated into English by NKF who is fluent in both Bahasa and English. The accuracy of translation was maintained through an iterative process of checking and rechecking transcripts against the translated interpretations to examine the meaning in both Bahasa (source) and English (translation) [51]. To maintain reliability and validity of the data, the final themes and interpretation presented in this paper, team-based analysis and discussion was also performed at regular supervision meetings. The team-based analysis and discussion was held throughout the data analysis process. For example, after the identification of the thematic framework and indexing or coding (step 2 and 3), data were presented at regular supervision meetings and discussed, and comments were provided for revision and improvement. Similarly, it was performed after the arrangement of the themes in a chart and throughout mapping and interpretation process (step 4 and 5), which resulted in the final themes and interpretation as presented in this paper. Data analysis was guided by the five steps of qualitative data analysis framework introduced in Ritchie and Spencer [52]. These steps include (i) *familiarisation* with the data through reading each transcript, breaking down the data into small chunks of data, and making comments or labels to the data; (ii) *identification of a thematic framework* by writing down key issues and concepts that recurrently emerged from the data; (iii) *indexing the data* by listing the open codes that had been made to the data to identify similar or redundant codes and reduce the long list of open codes to a manageable number, and then a closed coding was carried out to group similar codes under the same themes and sub-themes. The first theme was cultural practices of bride wealth, sanctions for spousal disputes and the purpose of marriage in Belu, which consisted of two sub-themes: (i) cultural practices of bride wealth and sanctions for spousal disputes and (ii) the purpose of marriage in participants’ culture. The second theme was Javanese cultural practices and Islamic religious beliefs about ‘ideal wife’, husband-wife relationships, sex and condom use, which comprised two sub-themes: (i) cultural practices and religious beliefs about ‘ideal wife’ or husband-wife relationships, and (ii) religious beliefs about sex and condom use. The themes and sub-themes were derived deductively based on prior knowledge and inductively as they emerged purely from the data; (iv) *charting* data through arranging appropriate thematic references in a summary of chart which enabled comparison across interviews and within each interview; and (v) *mapping and interpretation of the data* through which data were examined and interpreted [52]. These steps were used as they assisted us manage the qualitative data in a coherent and structured way, and guided the analytic process in a rigorous, transparent and valid way.

### Ethical consideration

Before commencing the interviews, participants were informed about the purpose of the study, the voluntary nature of their participation, their rights to withdraw without any consequences, and the approximate interview duration of 45 to 90 min. They were also advised that digital audio recording and notes would be undertaken during the interviews, and data or information they provided will be treated confidentially and anonymously by assigning a specific study identification letter and number for each of them (e.g., FP1, FP2, …., or MP1, MP2, …. FP stands for Female Participant and MP stands for Male Participant). They were also informed that that ethics approvals for study were solicited from Social and Behavioural Research Ethics Committee, Flinders University (No. 8286) and the Health Research Ethics Committee, Duta Wacana Christian University (No. 1005/C.16/FK/2019). All participants signed and returned an informed consent form on the interview day. Each participant received IDR 100,000 (±USD 7) reimbursement for transport and time after the interview.

## Results

### Sociodemographic profile of the participants

The participant’s age ranged between 18 to 60 years old, with the majority between 30 and 49 years (68 people). Half of them were (re)married and the others were unmarried (divorced, widowed or single). Several reported being diagnosed with any of these sexually transmitted infections: herpes, candidiasis, syphilis and gonorrhoea (13 people), and tuberculosis (28 people) (See Table 1). All (re)married women reported acquiring HIV transmission from their current or ex-husbands and all but four of them reported having HIV-positive current husbands. Similarly, all widowed, divorced and single/never married women but four reported acquiring HIV from their ex- or late husbands, or boyfriends. None of the widowed, divorced and single women reported engaging in any (sexual) relations following the diagnosis. Eighteen out of 25 (re)married men admitted transmitting the virus to their current or ex-wives and had HIV-positive current wives, while seven had HIV-negative current wives. Of all the widowers, single and divorced men, four reported actively engaging in sexual relations. The majority of participants graduated from senior and junior high school (55 people), and university (21 people), while the rest graduated from elementary school or dropped out of school. Both female and male participants engaged in different kinds of professions, while several women reported being housewives (21 people). Most participants in Yogyakarta were Muslim and the ones in Belu were Christians (see Table 1).

**Table 1:** Sociodemographic profile of the participants.

Characteristics	Women Living with HIV		Men Living with HIV	
	Yogyakarta (n = 26)	Belu (N = 26)	Yogyakarta (N = 20)	Belu (N = 20)
**Age**				
18–19		2		
20–29	6	4		7
30–39	12	12	10	5
40–49	8	6	10	5
50–59		1		2
60–69		1		1
**Marital status**				
Single	5	3	5	7
Divorced	5	1	2	
Widowed/r	3	12		1
(Re)Married	13	10	13	12
**HIV diagnosis**				
1–5 years ago	16	18	6	15
6–10 years ago	7	7	7	4
11–15 years ago	3	1	7	1
**Other infections**				
Herpes	2		2	1
Candidiasis	1	3		
Syphilis			2	
Gonorrhoea			2	1
TB	4	5	10	9
**Religion**				
Islam	23	1	17	
Catholic	2	25	3	19
Protestant	1			1
Hindu				
Other				
**Education**				
University graduate/Diploma	6	6	7	2
Senior High school graduate	13	5	11	8
Junior High school graduate	6	6	2	4
Elementary school graduate	1	8		6
Elementary school dropout		1		
**Occupation**				
Housewife	10	11		
Entrepreneur	3	6	10	1
Tailor	1			
Sex worker	1			
NGO worker	3			
Laundress	1			
Teacher				2
Farmer				3
Police				1
Nurse / health worker	1	2		
Shop keeper	1			
Private employee	2	2	5	1
Banker	1			
Retired civil servant		1		1
Civil servant		1		
University student	1			1
Taxi/truck/Motorbike taxi driver			1	8
Iron welder				2
Mechanic			1	
Unemployed	1	3	3	

### Cultural practices of bride wealth, sanctions for spousal disputes and the purpose of marriage in Belu

#### Cultural practices of bride wealth and sanctions for spousal disputes

Cultural practices of some tribes in Belu were indicated to play an important role in marriage and spousal relationships of the participants and contributed to HIV transmission among them, which did not exist in Javanese culture in Yogyakarta. For example, the cultural practice of a man and his family paying bride wealth to the bride’s family was reported by nearly half of the women and a few men whose tribes applied bride wealth in marriage and influenced their spousal (sexual) relationships. Payment of bride wealth seemed to put men or husbands in a position of power over women. Women were placed in the position where they were culturally obliged to obey and serve their husbands, including sexually, even when they knew of their husbands’ promiscuous behaviours. This imbalance of sexual power was a significant factor in HIV transmission from husbands to their wives:

*“There was bride wealth (from her late husband) because my father is from XXX (name of a tribe in Belu that applies bride wealth). After the bride wealth was received by my family I followed my husband and became a part of my husband’s family. Therefore, if my husband wanted anything …. including sexual demand, I just needed to fulfil it. My husband has already paid the bride wealth, so if we fought then where should I go? If I go to my parents, then they would send me back (to her husband). …. I knew that he (her late husband) visited the women (female sex workers) in XX (name of a brothel) but I could not continuously reject every time he asked (for sex) ….” (FP25, widowed, Belu)*.

*“Husband is the one who has the power in a household. So, a wife must listen to her husband as the one who has the power. A wife must serve her husband, including in husband-wife (sexual) relations. In our culture, if the bride wealth has been paid then the wife must submit to her husband, all the decision-making in the household is at the hand of the husband as the head of the household. I have paid the bride wealth, so my wife must submit to me as her husband and the head of the household” (MP11, married, Belu)*.

Several women in Belu talked about cultural norms and practices regarding sanctions for spousal dispute, since it is regarded as an embarrassment for a husband and wife and their respective families. Women expressed inability to ‘say no’ to sexual advances and felt they had no choice, but to oblige to husbands’ sexual demands to avoid verbal and physical disputes which could be embarrassing if known to other family members or neighbours and lead to cultural sanctions. The cultural sanctions require them to provide sacrificial animals and a dozen bottles of alcohol, which are used to serve traditional male leaders within a tribe, who gather to teach and provide cultural advice to the couples in disputes. Avoidance of these disputes, feelings of embarrassment and shame, and risk of public cultural sanctions was reported to prevent the women from questioning their husband’s risky sexual behaviour with other women, including sex workers:

*“I do not like (spousal) dispute. It is embarrassing if the neighbours hear about it. It is an embarrassment not only for us (the woman and her husband) but more for our parents, uncles and extended families…. If we (the woman and her husband) have a dispute verbally or physically and they know about it, we could get cultural sanction. We once had a cultural sanction: a pig and a dozen bottle of alcohol. So, I try not to get involved in spousal dispute. Thus, sometimes if he (her husband) wants (to have sex) then I just have to serve him even though I do not want to, or I am suspicious that he has had sex with other women or sex workers. It is because I do not want to fight and get cultural sanction” (FP18, married, Yogyakarta)*.

However, some married women and men in Belu also talked about respecting and listening to each other in their spousal (sexual) relationships and family decisions irrespective of their knowledge of the position of a husband as the head of a family. These were also described as a reflection of their understanding and acceptance of each other in spousal relationships:

*"Indeed, in our culture a husband is the head of the family, but my husband and I respect and listen to each other in our spousal relationship and family. My husband never imposes what he wants, including in terms of husband-and-wife intimate relationship. I think it is because we have been married for a long time and already have children, we understand and accept each other” (FP17, married, Belu)*.

*"In terms of husband-and-wife intimate relationship or decisions in our family*, *my wife and I listen to each other*. *We discuss things together*, *not only myself who makes decisions even though I am the head of the family*. *We respect each other*. *In our intimate relationship*, *we already know each other*, *if I want (sex) and my wife also wants*, *we know the signs*. *There has never been any pressure from me in sexual matter and I also understand the condition of my wife*, *both of us are (HIV) positive” (MP2*, *married*, *Belu*)

#### The purpose of marriage in participants’ culture

The purpose of marriage in the participants’ culture, which is to have children, was reported as a barrier to condom use practices among the participants in Belu. This was reflected in the stories of several women and men interviewed who described that they never thought of condom use due to the awareness of such purpose, which also influenced their own and their families’ expectations for them to have children:

*“My (late) husband and I wanted to have children, so (we) did not think of using condoms. In our culture here, getting married is to have children, and our family, especially parents, wanted to have grandchildren. So, it (condom use) was never in my mind even though I was aware (after the HIV diagnosis) that my husband had risky sexual behaviour” (FP9, widowed, Belu)*.

*“My wife and I never used condoms because I think the consequence of using condoms is that we will not have children. We get married to have children. This is our culture, getting married is to have children. The families will continuously ask ‘is your wife already pregnant?’ If we use condoms then this (pregnancy) will not happen” (MP2, married, Belu)*.

In addition, the majority of married men and several women in Belu perceived that condom use was not a common practice in marriage or passed down by their parents or ancestors. Such a perception seemed to also influence their condom use practices or support their consistent engagement in unprotected sex after the HIV diagnosis, a high-risk factor for further HIV transmission among husbands and wives:

*“Before I contracted HIV up to now, I never used condoms. . . . . Condom use is not a common practice in spousal relationships, I never heard of other people talking about the necessity of condom use in a husband-and-wife relationship” (FP7, remarried, Yogyakarta)*.

*“Condom use is not a common thing in a husband-and-wife marital relationships, I have never heard of any parents or ancestors talking about condoms. It also feels weird if I have to use a condom every time I have sex with my wife” (MP9, married, Belu)*.

### Javanese cultural practices and Islamic religious beliefs about ‘ideal wife’, husband-wife relationships, sex and condom use

#### Cultural practices and religious beliefs about ‘ideal wife’ or husband-wife relationships

Javanese cultural practices in marriage which have expectations about an ‘ideal wife’ and husband-wife relationships seemed to have an influence on spousal sexual relations for female and male participants in Yogyakarta, which were not identified from the participants in Belu. Half of the women and several men interviewed in this setting described Javanese culture of an ideal wife as a woman with characteristics including to serve, obey and listen to the husband, or obliged to respect, submit and does everything the husband wants, including sexually. Serving husbands sexually was described by the participants as part of women’s duty and a sign of loyalty to the husbands. Such cultural practices and expectations were lived experiences in these participants’ marriages or spousal sexual relations and seemed to also prevent some women from questioning their husbands’ sexual behaviours:

*“An ideal wife in Javanese culture is the one who serves her husband, submits to her husband, listens to her husband, does whatever her husband says, is not against her husband, and takes care of household chores, children and husband. I live these cultural values in my life as a wife” (FP15, married, Yogyakarta)*.

*“The culture in Yogyakarta or Java dictate for a wife to submit to her husband. A wife must do everything her husband says. It must be like that. A wife who submits to her husband is the one who serves her husband and her husband’s needs, including intimate (sexual) need. We live in our culture, so it should be like that” (MP6, remarried, Yogyakarta)*.

The Javanese cultural practices were similar to the religious beliefs in Islam about husband-wife sexual relationships. The stories of the majority of Muslim women and several men in Yogyakarta showed that religious beliefs played an important role in their sexual practices. These religious beliefs were not identified by the Christian participants in Belu. The Islamic religious beliefs such as ‘sexually serving a husband in marriage is a worship of a wife and refusing to do so is a sin and makes the angels angry’, seemed to shape their concepts about sex, their spousal sexual relations and practices in marriage, and contribute to HIV transmission among them:

*“My experience was that if my husband wanted (to have sex) then I served him. …. Even though I was not in the mood to have sex, …. I feared being a sinner because the (religious) thought says so and I obey it. It would be a big sin if I refused. Besides, it (serving husband sexually) is a worship of a wife” (FP11, Muslim woman, widowed, Yogyakarta)*.

*“In Islam, a wife must obey and treat her husband well. She must obey everything her husband said. Whatever a wife wants to do she must firstly get the permission from her husband. She must ask her husband as the head of the family, if her husband does not allow and she insists on doing it, then it is a mistake, a sin for her because she does not obey her husband, she is against her husband. It is also the same in sex matter, as a husband if I want to have sex then my wife must serve me, otherwise it is a sin for her. ….” (MP3, Muslim man, married, Yogyakarta)*.

However, several Muslim women explained that whilst they mostly followed their religious beliefs on husband-wife sexual relations, they adjusted such beliefs to their physical and emotional needs. Similarly, several Muslim men in the setting, who were also aware of these cultural practices and religious beliefs reported that they adapted such practices and beliefs to their wife’s condition and did not force their wife to completely adhere to these practices and beliefs. Good spousal communication and understanding were reported to help them adjust these practices and beliefs to their situation and lead to positive collaborative decision making in their spousal relationships:

*“In Islam, the one who can bring a wife to heaven is the husband. So, a wife who serves everything requested by a husband will be going to heaven. That is the way for a wife to heaven. …. If my husband asks to have sex but I am tired, then I will tell him kindly to do it tomorrow, do not refuse. So, there is communication” (FP12, Muslim woman, married, Yogyakarta)*.

*“The Javanese culture is very similar to the religious thoughts in Islam, a wife must listen, obey and submit to her husband. I do not do that in my family or my relationship with my wife because we understand each other. My wife and I complement each other. She does not have to do everything I want. We always talk to each other. For example, if I want something from her but she cannot do it because she is tired or not feeling well, she will tell me” (MP1, married, Yogyakarta)*.

#### Religious beliefs about sex and condom use

Islamic religious beliefs about sex were also reported to influence condom use behaviours of some unmarried Muslim female and male participants in Yogyakarta, which were not identified in the Christian interviewees in Belu. For example, Islamic religious beliefs that disallow pre-marital or non-marital sex, led their perceptions and fear on the possibility negative judgement and labelling from other people towards their engagement premarital sex practices, if known. Such perceptions and fear prevented them from buying and using condoms, which was a supporting their engagement in unprotected sex through which they acquired HIV. Narrative from an unmarried woman who was diagnosed with HIV several years ago illustrated such perceptions and fear:

*“It is also the same in religion (Islam) that people have to get married first and they can have sex after they are officially husband and wife. …. That is the reason I feel ashamed to buy condoms because if my neighbours see me buying condoms then I will be labelled negatively. They could label me as a sinner. …. There was not condom use at all in sex with my (ex and current) boyfriends, that is why I get this (HIV). (Her current boyfriend does not know about her HIV status)” (FP23, Muslim woman, single, Yogyakarta)*.

A conservative interpretation of Islamic religion regarding prohibition of condom use in marriage was reported by a number of Yogyakarta participants, who said it influenced their sexual behaviours, a factor which was not identified in the Christian participants in Belu. For example, several married Muslim women in this setting described that the lack of condom use in sexual relations with their husband was partly due to such reason, a factor that contributed to HIV transmission among them. The following quote from a 29-years-old Muslim woman who contracted HIV from her ex-husband several years ago illustrated such interpretation:

*“We (the woman and her ex-husband) did not use condoms because it is not allowed in religion (Islam). I got HIV from him because I know he previously married a female sex worker who is HIV positive (Her current husband is also HIV-positive)” (FP10, Muslim woman, remarried, Yogyakarta)*.

However, some Muslim male participants, whose wives were not infected with HIV, reported that religious beliefs in Islam that prohibit the use of condoms in husband-wife sexual relations did not influence their spousal sexual behaviours. They started using condoms consistently in their sexual relations with their wives following the HIV diagnosis even though they were aware that condom use is prohibited in Islam. Condom use practice was employed to prevent husband-to-wife HIV transmission:

*“I use condoms after being diagnosed with HIV because I am afraid of transmitting it to my wife. …. We (the man and his wife) know that condom use is not allowed in Islam, but this is for our benefit and health, my wife can get it (HIV) if I do not use condoms” (MP7, married, Yogyakarta)*.

*“In Islam, condoms and other contraception methods are not allowed, my wife and I are aware of that but after the diagnosis I always use condoms in sexual relation with my current wife because I am afraid of transmitting it to her” (MP19, remarried, Yogyakarta)*.

## Discussions

Despite a global reduction of HIV infections, new diagnoses in Indonesia have increased significantly over the last decade. This paper describes the influence of cultural practices and religious beliefs on sexual relationships, behaviours and practices of women and men living with HIV in Belu and Yogyakarta, as contributing factors for HIV transmission, which have been understudied, both in Indonesia and globally [27–30].

The current study findings suggest that women are vulnerable to HIV transmission due to cultural practices and norms, and strict religious beliefs that lead to power imbalance, including sexually. Cultural practices of bride wealth, and sanctions for spousal disputes in Belu, the expectations of an ‘ideal wife’ and sexual practices within husband-wife relationships in Javanese culture, all play a part in women being unable to be in control of their own bodies and health. Such norms and practices oblige women to obey, submit and serve their husbands, put men or husbands in a position of power over women, and shape men’s perceptions of their entitlement to be served by their wives, including sexually. As a consequence, women are disempowered and unable to negotiate and consent to their husbands having access to their bodies. These cultural practices also put married women in a vulnerable position to HIV infection, for example when they had to oblige to sexual demand of and unsafe sex with husbands, even when they suspected them to being engaging in extra-marital sexual behaviours with other women, such as female sex workers. Our findings show overall male dominance in both Belu and Yogyakarta, with occurrence of predominantly male-led sexual decision making in spousal sexual relations. These observations are in conformity with the findings of previous studies [23–25], that have associated male dominance in sexual matters and decision making with the lack of or low condom use by men or husbands, a supporting factor for HIV transmission among spouses or women. These are also in line with the constructs of the logical model for behavioural and environmental factors diagnosis, which suggests that socioenvironmental factors (e.g., in the case of this study, the influence of men, which is underpinned by cultural norms and practices, towards women’s sexual decision making and behaviour) determine or contribute to health problem or HIV transmission among women [37]. Similarly, the current findings indicate that these norms and practices imposed the fidelity of women or wives towards husbands as culturally expected, regardless of their knowledge or consent of the husbands’ risky sexual encounters with other women, such as female sex workers, another risk factor for HIV transmission among the women and their spouses. This is in line with previous findings of studies in Cambodia and Uganda and the constructs of the logical model [23, 24], suggesting that in some cultures males are accepted and expected to have multiple sex partners or more than one wife, while women are expected to remain faithful to their husbands.

The current findings show that the purpose of marriage which is to have children and condom use as an uncommon practice within marriage in culture in Belu, and Islamic religious beliefs that prohibit condom use in husband-wife sexual relations [53, 54], were supporting factors for unprotected sex among married participants across the study settings. It is therefore reasonable to allude that these factors contribute to HIV transmission within marriages, especially under circumstances that husbands engage in risky sexual behaviours with HIV high-risk women, such as female sex workers. We also suggest that it is not only the participants’ Islamic beliefs of premarital or non-marital sex which is forbidden [55, 56] that impacted their condom use behaviour. Participants also reported fear of stigma and social consequences of engaging in premarital sex as a driving force behind not using condoms. The findings support previous studies conducted elsewhere [57, 58], suggesting that a failure to adhere to religious beliefs leads to unprotected sex or risky sexual behaviours and HIV transmission. On the contrary, other studies [59, 60] have reported that following religious beliefs that emphasise premarital virginity or abstinence leads to lower levels of sexual risk among unmarried or young people through the decision to avoid high-risk sexual behaviours. However, the current findings also suggest that some participants adjusted these cultural practices and religious beliefs (e.g., on husband-wife sexual relations and condom use) to their own conditions due to the understanding of the importance of condom use for HIV prevention and for the sake of their health. These findings are consistent with previous suggestions that having a good knowledge of HIV transmission and prevention was a supporting factors for condom use [48, 61]. In addition, it should be acknowledged that Christian beliefs also forbid the use of contraception including condoms in marriage and premarital or extramarital sex [62]. However, the current study indicates that the Belu participants lacked knowledge about specific Christian beliefs around sex and condom use. This seemed to be the underlying reason for them not addressing in their comments the possibility of the influence of such religious thoughts on their sexual relations, practices and possibly HIV transmission among them.

The findings of the current study indicate that conservative Islamic religious beliefs about husband-wife sexual relations also influenced spousal sexual relations of married Muslim participants in Yogyakarta and seemed to contribute to husband-to-wife HIV transmission among them. The Islamic religious beliefs that sexually serving a husband in marriage is a worship of a wife and refusing to do so is a sin [63, 64], influenced the perceptions of some married women and men about sex, and their sexual relations and practices in marriage. Such an influence was reflected in women’s inability to refuse husbands’ sexual demands regardless of their desire, women’s willingness to oblige to husbands’ sexual demands due to the fear of committing a sin, and men’s perceptions that they are entitled to sexual services by their wives. Such religious beliefs also seemed to be the supporting factors for male dominance over women with regards to spousal sexual decision making. The current findings provide strong evidence that cultural and religious factors did influence the likelihood of the female participants being infected with HIV, especially those who reported acquiring the infection from their current, former or late husbands. It is also plausible to suggest that these factors also increase the likelihood of exposure to co-infection and other STI’s, by the male participants and indeed, males in general in these study settings, from their HIV-positive spouses or partners.

### Limitations and strengths of the study

The findings should be interpreted with some caution due to several limitations. In general, the study involved a small number of participants in each study setting, thus the study results, including cultural and religious aspects presented in this paper reflected specific situations, views and experiences of the participants. The use of snowball sampling technique may have also led to the recruitment of participants from the same networks, could have under sampled PLHIV who were outside of the social networks of the current participants and led to incomplete overview of the experiences and perceptions of PLHIV about the topics being investigated. Besides, the involvement of widows, single and divorced participants have led to them not being able to provide information on their current experiences of spousal sexual relations due their non-marital status, hence this is also a limitation which should be considered once interpreting the findings of this study. Finally, single coder might also be a limitation in this study since it limited the triangulation during the coding process, although team-based analysis and discussion was conducted. However, to the best of our knowledge, this is first qualitative inquiry that explores cultural and religious factors associated with the transmission of HIV infection in the context of Indonesia. Thus, these findings are useful for the development of HIV programs or interventions that take into consideration cultural and religious aspects to improve knowledge, understanding and health promoting behaviours of PLHIV and general community members in the study settings and other settings in Indonesia and globally.

## Conclusions

Our findings suggest that cultural practices on bride wealth, sanctions towards spousal dispute, purpose of marriage, condom use and cultural expectation of an ‘ideal wife’ are contributors to HIV transmission among both women and men living with HIV in Belu and Yogyakarta. Similarly, religious beliefs about spousal sexual relationships or women’s obligation to serve husbands, forbidden premarital sex and participants’ individual of interpretation of their religious beliefs about condom use in husband-wife sexual relations, do contribute to HIV transmission among them. There is an urgent need for HIV education and policy change, that address and challenges cultural practices and religious thoughts on sex, condom use and spousal sexual relationships. This change should be tailored for specific population groups, especially women, but also religious groups, and communities, to dispel myths about HIV, enhance understanding about condom use and their protective function, and encourage condom use practices. Most importantly, a change is needed in the form of female empowerment, and challenging the patriarchal system that these women live in, if HIV transmission is truly going to be impacted. The findings also indicate the need for involvement of religious leaders in HIV education programs to bring insight to people and help them interpret religious beliefs or thoughts in health promoting ways. Further, large scale studies are needed that deeply explore aspects of culture and religion and how these are contributing to an HIV epidemic among both women and men in Indonesia and elsewhere.

## Supporting information

S1 Checklist COREQ checklist. (DOCX)

S1 File Interview guide. (DOCX)
