# Supplementary material for: Cultural and religious determinants of HIV transmission: A qualitative study with people living with HIV in Belu and Yogyakarta, Indonesia
Source: PLoS One. 2021 Nov 15;16(11):e0257906. doi: 10.1371/journal.pone.0257906 (PMC8592403; doi:10.1371/journal.pone.0257906)
Supplement: S1 File — (DOCX) [file pone.0257906.s002.docx]

**Interview questions:**

What do you think about husband-wife relationship in your culture?

- Are there any culture practices or norms that govern husband-wife relationship in your culture? Tell me more about it.
- Are any cultural values that are highly upheld by women and men in marriage? Tell me more about it.
- What do you think about rights and power/position of husband and wife in marriage from the perspectives of your culture? Please explain about it.
- What is the purpose of marriage in you culture? Please explain.
  - How does it guide or influence your spousal relationships, if any?

Would mind describing about condom use in your culture? Any practices or perceptions related to it.

- Do you think they influence your sexual behaviours? Why? Please tell me more.

What are the perspectives in your culture about premarital or extramarital sexual relation in your culture? If any, please tell me more about it.

What do you think about husband-wife relationship in your religion?

- Are there religious thoughts and values that govern husband-wife relationship? Tell me more about it.
- Are there religious values that are highly upheld by women and men in marriage? Tell me more about them.
- Based on your religious thoughts, norms and values: how should husband and wife behave towards each other in marriage?
  - In what matters? Please explain about it.
- Based on your religious thoughts and norms, when it comes to decision about spousal sexual relations: who makes the decision? Tell me more about it.
  - How do those thoughts, if any, influence your spousal sexual relations? Please tell me some experience of yours.
  - Have you ever thought of or refused sex with your partner of husband due to some reason? Tell me more about it.

What do you know about the thoughts in your religion in relation to condom use?

- - Do you think they influence your sexual behaviours or condom use practices? Tell me about it.

What are the religious thoughts in relation to premarital or extramarital sexual relation?

- - How do they influence your sexual behaviours? Why?
